# Epidemiology of human leptospirosis in urban and rural areas of Brazil, 2000–2015

**DOI:** 10.1371/journal.pone.0247763

**Published:** 2021-03-04

**Authors:** Deise I. Galan, Amira A. Roess, Simone Valéria Costa Pereira, Maria Cristina Schneider

**Affiliations:** 1 Department of Environmental and Occupational Health, The George Washington University, Washington, District of Columbia, United States of America; 2 Department of Global and Community Health, George Mason University, Fairfax, Virginia, United States of America; 3 Secretary of Health Surveillance, Ministry of Health of Brazil, Brasilia, Federal District, Brazil; 4 Department of International Health, Georgetown University, Washington, District of Columbia, United States of America; 5 Institute of Studies in Collective Health, Federal University of Rio de Janeiro, Rio de Janeiro, Rio de Janeiro, Brazil; University of Minnesota, UNITED STATES

## Abstract

**Background:**

Leptospirosis is one of the most widespread zoonosis in the world and Brazil has the highest number of cases in Latin America. Transmission occurs mainly through exposure to water and soil contaminated by the urine of infected animals. The goals of this study are to describe the geographic distribution, demographic characteristics and exposure factors of urban and rural cases of leptospirosis, and identify spatial clusters in urban and rural areas of Brazil.

**Methods/results:**

A retrospective epidemiological study was carried out using 16 years (2000–2015) of surveillance data from the Brazilian Ministry of Health. Cases were described by age, sex and race, and exposure factors were characterized in urban and rural areas. A spatial autocorrelation analysis was conducted using local Moran’s I to identify urban and rural clusters of disease. On average 3,810 leptospirosis cases were reported annually with higher numbers in urban areas. National urban and rural incidence rates were the same (1.9 cases/100,000 population), however, regional differences were observed. Urban incidence rates were higher in the North and Northeast regions, while rural incidence rates were higher in the Southeast and South. The main exposure factor reported in urban and rural areas was exposure to places with signs of rodents, followed by flood in urban areas and agriculture and animal farming in rural areas. Clusters of leptospirosis were identified in densely populated urban areas of the North, Southeast and South regions, while rural clusters were concentrated in of the Southern region with large agriculture and animal farming practices.

**Conclusions:**

This study highlights that leptospirosis is an important public health problem in both urban and rural areas of Brazil. The results provide decision-makers with detailed information about where disease incidence is high and can be used in the development of prevention and control strategies for priority areas and risk groups.

## Introduction

Leptospirosis is one of most geographically widespread zoonosis, however, it remains a neglected disease [1–3]. Leptospirosis is caused by a pathogenic spirochete bacterium of the genus *Leptospira*, and is transmitted mainly through exposure to water and soil contaminated by the urine of infected animals [4, 5]. The greatest burden of disease is among resource-poor populations in tropical regions, mostly in low- and middle-income countries [1, 6]. Worldwide, leptospirosis is estimated to cause 1.03 million cases and 58,900 deaths each year [1]. In the region of the Americas, official surveillance reports estimate approximately 10,000 annual human cases, with 95% of them in Latin America, among which 40% are reported in Brazil [7].

The transmission pathways of leptospirosis in the environment are complex and have been reported in a variety of settings, from large urban centers, to remote rural areas [8–11]. Several outbreaks have been reported in urban areas around the world after heavy rainfall and flood events, which bring the bacteria and their animal hosts in closer contact with humans, including in Guyana, India, Indonesia, Italy, Philippines and Malaysia [12–17]. A systematic review of leptospirosis outbreaks identified that out of 318 leptospirosis outbreaks from 1970 to 2012, among the ones that had information about the settings, 16% occurred in urban and 15% in rural areas [18]. Endemic transmission of leptospirosis is reported in rural areas and is frequently associated with specific occupational groups therein, as described in Argentina, Brazil, Nicaragua, Lao, Mexico, New Zealand and Sri Lanka [19–27].

In Brazil, the notification of leptospirosis cases is mandatory since 2000 and surveillance is conducted across the country within the purview of the Ministry of Health [28]. Brazil has a laboratory network for leptospirosis diagnosis composed of state laboratories that perform enzyme-linked immunosorbent assay (ELISA-IgM), five regional laboratories that also perform serological testing using the microagglutination test (MAT) and one national reference laboratory that performs all tests, including PCR and isolation of *Leptospira* [7, 29]. Investigations of deaths attributable to leptospirosis are conducted using immunohistochemistry and/or positive polymerase chain reaction (PCR) [28].

Previous studies conducted in Brazil have demonstrated the importance of the disease in both urban and rural areas of the country [30–32]. In urban settings, leptospirosis is a major public health problem, due to rapid and spatially disorganized urbanization, inadequate sanitation and poverty, typically occurring in urban slums [8, 33, 34]. Large outbreaks are often detected after floods, and studies conducted in major urban centers like São Paulo, Rio de Janeiro, Salvador and Recife have shown that urban outbreaks occur due to low-income high-density populations living at the edge of streams and, in places with poor health infrastructure and rodent infestations [8, 35–40]. Leptospirosis also affects rural populations in Brazil, with one study conducted in the state of Rio Grande do Sul reporting up to eight times higher risk in rural areas compared to urban ones [20]. Subsistence farmers and rice field workers have been identified as high risk groups in rural areas of Brazil in previous studies conducted in the states of Rio Grande do Norte, Rio Grande do Sul and Rondônia [21, 41, 42].

Although several leptospirosis studies have been conducted in Brazil, there is a lack of a comprehensive exploration of the urban and rural patterns of the disease over an extended time period. In addition, important knowledge gaps remain in our understanding of the epidemiology of leptospirosis in remote and sparsely populated areas of Brazil. Thus, the goals of this study are to describe the geographic distribution, the demographic characteristics, the exposure factors of urban and rural cases of leptospirosis, and to identify spatial clusters of the disease in urban and rural areas of Brazil from 2000 to 2015. The results of this study will provide policy makers with detailed information about the spatial distribution and exposure factors for the disease in the last 16 years, since notification of human leptospirosis became mandatory in Brazil. These results can be used in the development of prevention and control actions or urban or rural priority areas.

## Material and methods

### Study area

Brazil is the largest country in South America and the fifth largest country in the world by both area and population [43]. According to the most recent census conducted in 2010, Brazil had a population of 190.8 million spread across 26 states and the federal district, with the majority of people living within 300 km of the coast and more than 83% in urban centers [44]. The country is divided into five regions: North, Northeast, Southeast, South and Center-West. Due to Brazil’s large territorial extension and varied topography, the climate varies considerably from region to region, but most of the country has a tropical or sub-tropical climate with an average annual rainfall between 1,000 and 1,500 mm [45]. The country has a diverse economy; the service sector is the largest one responsible for 67% of the GDP, followed by the industrial (27.5%) and agricultural sectors (5.5%) [46].

### Surveillance data and case definitions

Leptospirosis surveillance data from 2000 to 2015 was obtained from the Brazilian Ministry of Health (MOH) Citizen Information System [47]. Since 2000, notification of human leptospirosis cases is mandatory through the Ministry of Health’s Information System for Notifiable Diseases (acronym in Portuguese SINAN), through a passive surveillance system. Healthcare professionals are trained and required to complete a notification form if a patient is suspected of leptospirosis [48]. The notification form collects detailed information about the patients’ demographic information, epidemiological history including possible place of exposure and risk situations, clinical signs and symptoms, laboratory confirmation results and final disease classification (confirmed or discarded). In 2006, the notification form was updated to include minor modifications and it became effective across the country in 2007.

The Brazilian MOH defines a suspected case of leptospirosis as an individual with fever, headache and myalgia with either 1) potential exposure to environmental, occupational or other risk situation in the thirty days prior to the onset of the first symptoms, or 2) presence of one or more of the following signs or symptoms: conjunctival suffusion, signs of acute renal failure, jaundice and/or high levels of bilirubin and hemorrhagic phenomena [28]. Case investigation is conducted by the local authorities (at the county level) then forwarded to the state and national level [7].

Suspected cases are confirmed by one of two criteria: clinical/laboratory or clinical/epidemiologic. Clinical/laboratory confirmed cases are defined as having the presence of compatible clinical signs and symptoms associated with one or more of the following laboratory test results: 1) reagent enzyme-linked immunosorbent assay (ELISA-IgM) test with microagglutination test (MAT) seroconversion (two samples); 2) four-fold or greater increase in antibody titer by MAT (two samples) or one sample with titer equal to or greater than 800 by MAT; 3) isolation of *Leptospira* from blood; or 4) positive polymerase chain reaction (PCR) or immunohistochemistry for leptospirosis in suspected patients who eventually die [28]. Clinical/epidemiologic confirmed cases are defined as: 1) suspected cases who present with fever and changes in liver, renal or vascular function; and 2) who were exposed to potential risk factors; however, for some reason, material for specific laboratory tests was not collected or the result was non-reagent [28].

For the purpose of this study both clinical/laboratory and clinical/epidemiologic leptospirosis cases confirmed by the health authorities were included in the analysis, following the Brazilian MOH definition described above (hereafter referred to as cases).

### Data collection from study population

The 2000–2015 surveillance data were officially requested by the investigators and de-identified by the MOH. According to the lead researcher’s institution’s IRB, this study did not meet the definition of human subject research and did not require ethical approval since it did not involve any interaction with human subjects and the data provided by the Brazilian MOH were completely de-identified before furnished to the researchers. Demographic variables included in the analysis were:

**State of residence:** First subnational administrative level where the case reside.
**County of residence:** Second subnational administrative level where the case reside.
**Area of residence:** Zone of residence of case (urban or rural). Because no clear definition of peri-urban area of residence was found in the literature, the peri-urban classification was not included in the analysis (a total of 789 confirmed cases).
**Sex:** Male and Female.
**Age:** Age was used as a continuous and categorical variable with age classes (0–5, 6–14, 15–24, 25–39, 40–59, >60 years old).
**Race:** White, black, East Asian ancestry, mixed-race or indigenous.
**Exposure factors included in the analysis were:** Self-reported exposure to one or more risk situations in the thirty days prior to the onset of the first symptoms reported by the healthcare professional in consultation with the suspected leptospirosis patient, indicating for each variable whether or not the patient presume exposure occurred. The notification form included exposure to: 1) water or mud from flood; 2) river, stream, pond or reservoir; 3) animal farming; 4) agriculture; 5) grain storage (data available since 2007); 6) place with signs of rodents (data available since 2007); 7) direct contact with rodents; 8) wasteland (data available since 2007); 9) garbage; 10) water tank; and 11) septic tank, grease trap or sewage.
**Date of notification:** Month and year when case was notified to the Ministry of Health.
**Population:** Data was obtained from the National Institute of Statistic of Brazil (Portuguese acronym: IBGE) [44]. The latest Brazilian Census was conducted in 2010 and information from this year was used in the study since it provided disaggregated population by demographic characteristics and geographic region closest to middle of the study period (2000–2015).

### Study design and statistical analysis

A retrospective epidemiological study was carried out using 16 years of leptospirosis surveillance data. Leptospirosis confirmed cases (in urban and rural areas) were described by time, space and demographic characteristics. The total number of cases (observations) varied for each variable according to its completeness in the database. Mean annual incidence rates were calculated at the national, regional and state level based on the number of cases over the study period per 100,000 population.

Demographic characteristics of human leptospirosis cases in Brazil were described by age, sex, race by urban and rural areas from 2000 to 2015. Chi-square was used to detect statistically significant differences between urban and rural areas [49]. Exposure factors were analyzed based on the number of cases that reported exposure to one or more risk situations. Visual exploratory analysis was conducted to present the differences between urban and rural areas for demographic variables and exposure factors.

County-level incidence rates were mapped for the entire country and for urban and rural areas. To reduce inflation of incidence rates due to small population sizes, the Empirical Bayes smoothing method was applied [50]. Local indicators of spatial association (LISA) were used to identify spatial clusters of leptospirosis at the county level by calculating local Moran’s I using smothered incidence rates between a given county and the average neighbors [51]. This spatial cluster analysis was done to identify concentrations of neighboring counties with a high incidence of leptospirosis considering their neighboring counties. Counties with high incidence rates of leptospirosis that are surrounded by other counties also with high incidence are classified as High-High. Low-Low clusters are counties with low values of leptospirosis incidence surrounded by counties with also low incidence. Spatial outliers are identified as High-Low and Low-High indicating counties with high or low incidence rates surrounded by counties with low or high incidence [51]. Statistical analyses were conducted using Stata 13. Associations at a p-value of 0.05 were considered statistically significant. Empirical Bayes smoothed rates were calculated using GeoDa vs 1.14 and LISA maps were constructed using ArcGIS vs 10.8.

## Results

### Geographic distribution

**Fig 1:**
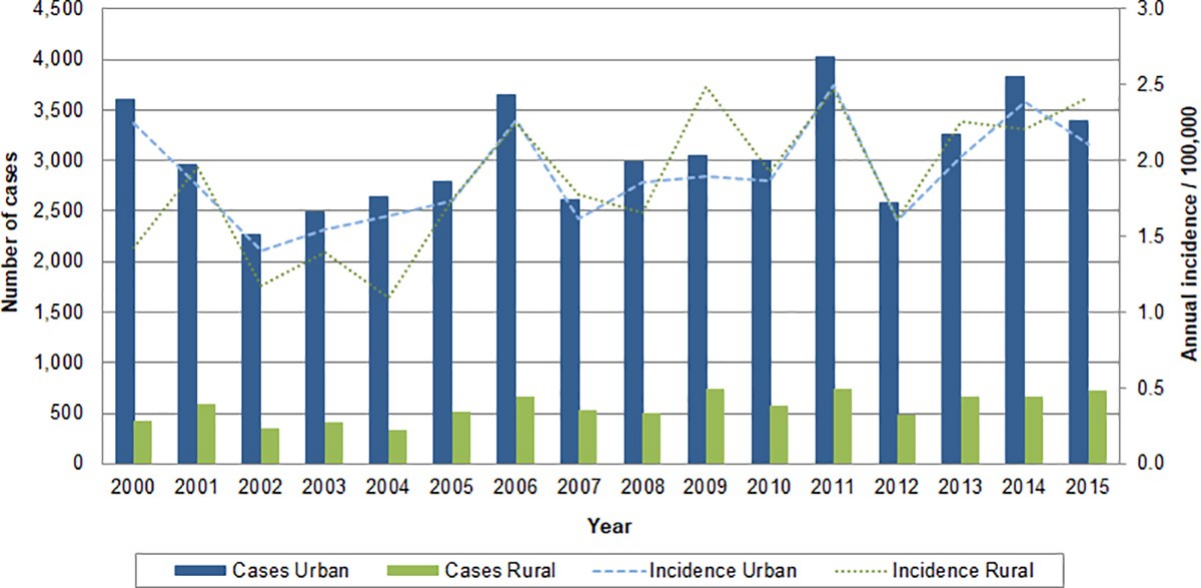
Number of cases and incidence rates of leptospirosis per 100,000 inhabitants in urban and rural areas by year, Brazil, 2000–2015.

The Brazilian Ministry of Health (MOH) registered a total of 248,616 suspected cases of leptospirosis from 2000 to 2015, of which 60,999 were confirmed based on the MOH national case definition criteria [52]. For the purpose of this study, 60,952 cases of leptospirosis were analyzed (47 observations were excluded due to insufficient information). On average, 3,810 confirmed cases of leptospirosis were reported annually during the study period, ranging from 2,739 cases in 2002 to 5,007 cases in 2011 (Fig 1). The country mean annual incidence rate for the study period was 1.9 cases per 100,000 population, ranging from 1.6 (in 2002) to 2.6 cases (in 2000 and 2011) per 100,000 population. Among the cases that reported information related to the disease outcome (n = 58,986), there were a total of 5,902 deaths reported in the study period with a case fatality rate of 10.0%. The period of the year with the highest number of cases was from January to March (S1 Fig).

Over the study’s 16-year period, 95.3% of cases (n = 58,083) reported area of residence, with urban number of cases predominantly higher than rural. The mean annual incidence rate in urban and rural areas was 1.9 cases per 100,000 population. Across the study period, the rural annual incidence was higher than or equal to the urban in 9 out of 16 years. The year 2011 had the highest number of reported cases with 4,025 urban and 737 rural, and both urban and rural annual incidence rates were 2.5 cases per 100,000 population. Brazil had the lowest number of cases of leptospirosis in 2002, with 2,270 urban cases and 351 rural cases, accounting for annual incidence rates of 1.4 and 1.2 cases per 100,000 population respectively.

All five regions and the 27 states, including the Federal District, reported cases of leptospirosis during the study period (Table 1). The Southeast, the most populated region in Brazil, reported the highest number of cases (19,705 cases corresponding to 33.9% of the total), followed very closely by the South region (18,477 cases). Throughout the country, cases were predominately urban (84.7% of reported cases) and concentrated in the Southeast and South; however, among the rural cases, more than 55% were reported in the South region. Analyzing incidence rates by region, the urban incidence was 3.8 and 2.5 times higher than the rural in the Northeast and North regions, respectively. The opposite was observed in the South region, where rural incidence was two times greater than the urban.

**Table 1:** Population, number of cases and incidence rates of leptospirosis per 100,000 inhabitants in urban and rural areas by region and state, Brazil, 2000–2015.

Region	Population	Cases of Leptospirosis *(n = 58*,*083)*		
State		Urban n (%)	Rural n (%)	Total n
		Incidence Rate	Incidence Rate	Incidence Rate
**North**	**15,864,454**	**7,434 (100.0)**	**1,076 (100.0)**	**8,510 (100.0)**
		**4.0**	**1.6**	**3.4**
Acre	733,559	3,089 (41.5)	519 (48.1)	3,608 (42.4)
		36.3	16.1	30.7
Amapá	669,526	1,201 (16.2)	50 (4.6)	1,251 (14.7)
		12.5	4.6	11.7
Amazonas	3,483,985	776 (10.4)	60 (5.6)	836 (9.8)
		1.8	0.5	1.5
Pará	7,581,051	1,985 (26.7)	193 (17.9)	2,178 (25.6)
		2.4	0.5	1.8
Rondônia	1,562,409	335 (4.5)	236 (21.9)	571 (6.7)
		1.8	3.6	2.3
Roraima	450,479	20 (0.3)	5 (0.5)	25 (0.3)
		0.4	0.3	0.3
Tocantins	1,383,445	28 (0.4)	13 (1.2)	41 (0.5)
		0.2	0.3	0.2
**Northeast**	**53,081,950**	**9,603 (100.0)**	**984 (100.0)**	**10,587 (100.0)**
		**1.5**	**0.4**	**1.2**
Alagoas	3,120,494	1,107 (11.5)	84 (8.5)	1,119 (11.2)
		3.0	0.6	2.4
Bahia	14,016,906	2,044 (21.3)	93 (9.4)	2,137 (20.2)
		1.3	0.1	1.0
Ceará	8,452,381	815 (8.5)	376 (38.2)	1,191 (11.2)
		0.8	1.1	0.9
Maranhão	6,574,789	346 (3.6)	124 (12.6)	470 (4.4)
		0.5	0.3	0.4
Paraíba	3,766,528	249 (2.6)	35 (3.6)	284 (2.7)
		0.5	0.2	0.5
Pernambuco	8,796,448	4,216 (43.9)	150 (15.2)	4,366 (41.2)
		3.7	0.5	3.1
Piauí	3,118,360	18 (0.2)	10 (1.0)	28 (0.3)
		0.1	0.1	0.1
Rio Grande do Norte	3,168,027	188 (2.0)	55 (5.6)	243 (2.3)
		0.5	0.5	0.5
Sergipe	2,068,017	620 (6.5)	57 (5.8)	677 (6.4)
		2.5	0.7	2.0
**Southeast**	**80,364,410**	**17,929 (100.0)**	**1,776 (100.0)**	**19,705 (100.0)**
		**1.5**	**2.0**	**1.5**
Espírito Santo	3,514,952	1,838 (10.2)	767 (43.2)	2,605 (13.2)
		3.9	8.2	4.6
Minas Gerais	19,597,330	1,192 (6.6)	241 (13.6)	1,433 (7.3)
		0.4	0.5	0.5
Rio de Janeiro	15,989,929	3,629 (20.2)	154 (8.7)	3,783 (19.2)
		1.5	1.8	1.5
São Paulo	41,262,199	11,270 (62.9)	614 (34.6)	11,884 (60.3)
		1.8	2.3	1.8
**South**	**27,386,891**	**13,523 (100.0)**	**4,954 (100.0)**	**18,477 (100.0)**
		**3.6**	**7.5**	**4.2**
Paraná	10,444,526	3,631 (26.8)	665 (13.4)	4,296 (23.2)
		2.5	2.7	2.6
Rio Grande do Sul	10,693,929	5,076 (37.5)	2,808 (56.7)	7,884 (42.7)
		3.5	11.0	4.6
Santa Catarina	6,248,436	4,816 (35.6)	1,481(29.9)	6,297 (34.1)
		5.7	9.3	6.3
**Central-West**	**14,058,094**	**687 (100.0)**	**117 (100.0)**	**804 (100.0)**
		**0.3**	**0.5**	**0.4**
Federal District	2,570,160	328 (47.6)	54 (46.2)	382 (47.4)
		0.8	3.8	0.9
Goiás	6,003,788	190 (27.6)	24 (20.5)	214 (26.6)
		0.2	0.3	0.2
Mato Grosso	3,035,122	76 (11.1)	25 (21.4)	101 (12.6)
		0.2	0.3	0.2
Mato Grosso do Sul	2,449,024	93 (13.5)	14 (12.0)	107 (13.3)
		0.3	0.2	0.3
**Total**	**190,755,799**	**49,176**	**8,907**	**58,083**
		**1.9**	**1.9**	**1.9**

* Population from 2010 Census [44].

There is a large range of the number of leptospirosis cases by state in Brazil, from 11,884 cases in the state of São Paulo (the most populous state), corresponding to 20.5% of the all cases in the country, to 28 cases in Piauí. Incidence rates in urban and rural areas also varied by state, with 13 out of the 27 states presenting higher incidence in rural versus urban areas, mainly in the South and Southeast regions. Incidence outliers were detected in urban areas of the states of Acre and Amapá, and in rural areas of the states of Acre, Rio Grande do Sul and Santa Catarina (S2 Fig). Acre presented the country’s highest urban and rural incidence rates (36.3 and 16.1 cases per 100,000 population, respectively).

A total of 2,843 (51.1%) out of 5,567 counties in Brazil reported at least one case of leptospirosis during the study period (ranging from 1 to 4,004 cases). In urban areas a total of 2,215 counties (39.8%) reported leptospirosis cases (ranging from 1 to 3,841 cases) and in rural areas a total of 1,176 counties (21.1%) reported at least one case (ranging from 1 to 356 cases). Cases were concentrated in the coastal areas of the South and Southeast regions, and in large counties (by surface area) of the North region. When disaggregated by urban and rural areas, higher incidence rates are seen in large urban centers in the coastal area of the South and Southeast region, and in the states of Acre and Amapá in the North region, with high rural incidence rates dispersed along the rural areas of the Southern states and in the state of Acre (S3 Fig).

### Demographic characteristics

**Fig 2:**
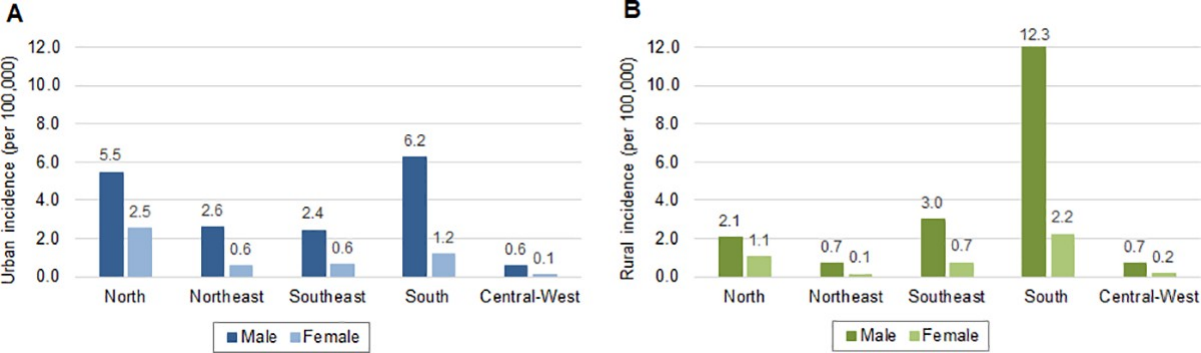
Incidence rates of leptospirosis per 100,000 inhabitants by sex, region and area of residence: (A) urban and (B) rural, Brazil, 2000–2015.

Throughout the 16-year period, there were significant differences (p<0.001) between urban and rural cases by sex, age and race (Table 2). Leptospirosis cases were predominantly in males (79.2%) in both urban (78.5%) and rural (82.9%) areas. Nationally, across males and females, the incidence rates of leptospirosis were similar, with incidence in males approximately 4 times higher than that in females (total, urban and rural). However, when analyzing the regional incidence rates by sex and residence area, differences were noted. The South region presented the highest incidence rates of leptospirosis in males in both urban (6.2 per 100,000 inhabitants) and rural areas (12.3 per 100,000 inhabitants) (Fig 2). In the North and Northeast regions, leptospirosis incidence rates in males were approximately 2.5 to 4 times higher in urban compared to rural areas, but the opposite was observed in the South region with the highest regional incidence detected in males in rural areas (12.3 per 100,000 inhabitants). Among females, the highest incidence was detected in the urban areas of the North region (2.5 per 100,000 inhabitants), while the lowest incidence occurs in both urban and rural areas of the Central-West region. Even though incidence among females was usually higher in urban areas, in the South region incidence was approximate two times higher in rural areas (2.2 per 100,000).

**Table 2:** Population, number of cases and incidence rates of leptospirosis per 100,000 inhabitants in urban and rural areas by demographic characteristics, Brazil, 2000–2015.

Characteristics	Population	Cases of Leptospirosis			p-value
		Urban n (%)	Rural n (%)	Total n (%)	
		Incidence Rate	Incidence Rate	Incidence Rate	
**Sex**					<0.001
*(n = 58*,*051)*					
Female	97,348,809	10,547 (21.5)	1,525 (17.1)	12,072 (20.8)	
		0.8	0.7	0.8	
Male	93,406,990	38,601 (78.5)	7,378 (82.9)	45,979 (79.2)	
		3.1	2.9	3.1	
**Age Classes**					<0.001
*(n = 56*,*193)*					
0–5	16,729,284	774 (1.6)	118 (1.3)	892 (1.6)	
		0.4	0.2	0.3	
6–14	29,204,713	4,368 (9.2)	720 (8.2)	5,088 (9.1)	
		1.2	0.8	1.1	
15–24	34,240,666	10,099 (21.3)	1,654 (18.9)	11,753 (20.9)	
		2.2	1.9	2.1	
25–39	46,735,171	14,902 (31.4)	2,678 (30.5)	17,580 (31.3)	
		2.3	2.6	2.4	
40–59	43,263,415	13,831 (29.2)	2,859 (32.6)	16,690 (29.7)	
		2.3	2.9	2.4	
>60	20,582,551	3,453 (7.3)	737 (8.4)	4,190 (7.5)	
		1.2	1.4	1.3	
**Race**					<0.001
*(n = 42*,*884)*					
White	90,621,281	17,843 (50.5)	5,194 (69.1)	23,037 (53.7)	
		1.4	3.0	1.6	
Black	14,351,162	2,500 (7.1)	283 (3.8)	2,783 (6.5)	
		1.3	0.9	1.2	
East Asian	2,105,353	260 (0.7)	47 (0.6)	307 (0.7)	
		0.9	1.0	0.9	
Mixed	82,820,452	14,683 (41.5)	1,954 (26.0)	16,637 (38.8)	
		1.4	0.8	1.3	
Indigenous	821,501	79 (0.2)	41 (0.5)	120 (0.3)	
		1.5	0.5	0.9	

The median age of leptospirosis cases across the study period was 33.8 years. In both urban and rural areas, approximately 82% of cases of leptospirosis were predominately among those in the economically productive age groups, from 15 to 59 years of age and this group also had the highest incidence compared to other age groups. The median age was slightly higher in rural areas (35.8 years old) compared to urban areas (33.4 years old). By region, there were little differences observed in age between urban and rural areas, with slightly older cases in rural areas of the South region (median age = 38.9 years old) (S4 Fig).

In both urban and rural areas, cases were mostly white (53.7%), followed by mixed-race (38.8%), however a higher percentage of white cases were among those in rural (69.1%) compared to urban areas (50.5%). Analyzing leptospirosis incidence by race, white in rural areas showed the highest incidence at 3.0 per 100,000 population. The regional distribution of leptospirosis in urban and rural areas by race showed similar distribution of cases in each region, with the South and North regions presenting the highest percent of white and mixed-race, respectively (S5 Fig). A higher percentage of leptospirosis cases among indigenous populations was seen more frequently in rural areas of the Central-West (2.5%) and North regions (2.2%).

### Exposure factors

**Fig 3:**
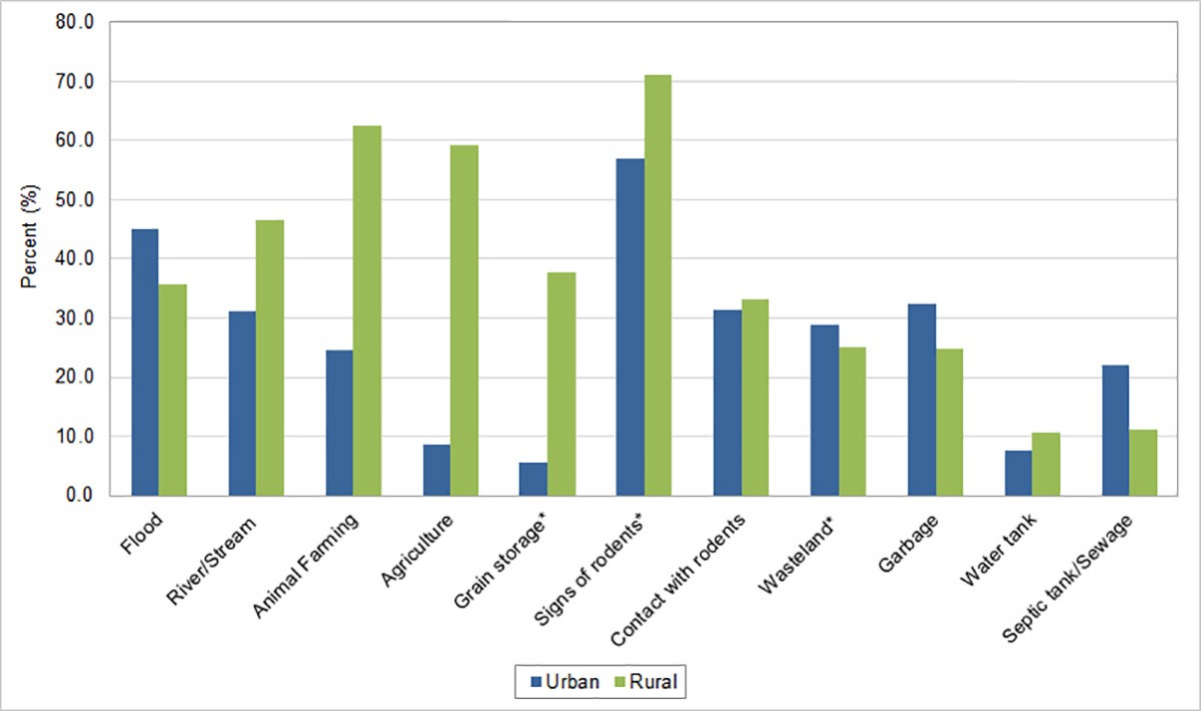
Percentage of cases of leptospirosis by exposure factors in urban and rural areas, Brazil, 2000–2015. *Data available after 2007.

Among leptospirosis cases, exposure to places with signs of rodents was the most often identified exposure factor in both urban (56.9%) and rural areas (71.2%) (Fig 3 and S1 File). Animal farming (64.2%), agriculture (59.2%), river/stream (46.7%) and grain storage (37.8%) exposures were predominantly found among rural cases. Flood was the second most reported exposure factor in urban areas (45.2%), but also important for rural areas (35.7%). Exposures to septic tanks were almost two times higher in urban (21.9%) compared to rural areas (11.2%), while direct contact with rodents and exposure to wasteland, garbage and water tank were reported similarly in both urban and rural areas.

### Spatial clusters

**Fig 4:**
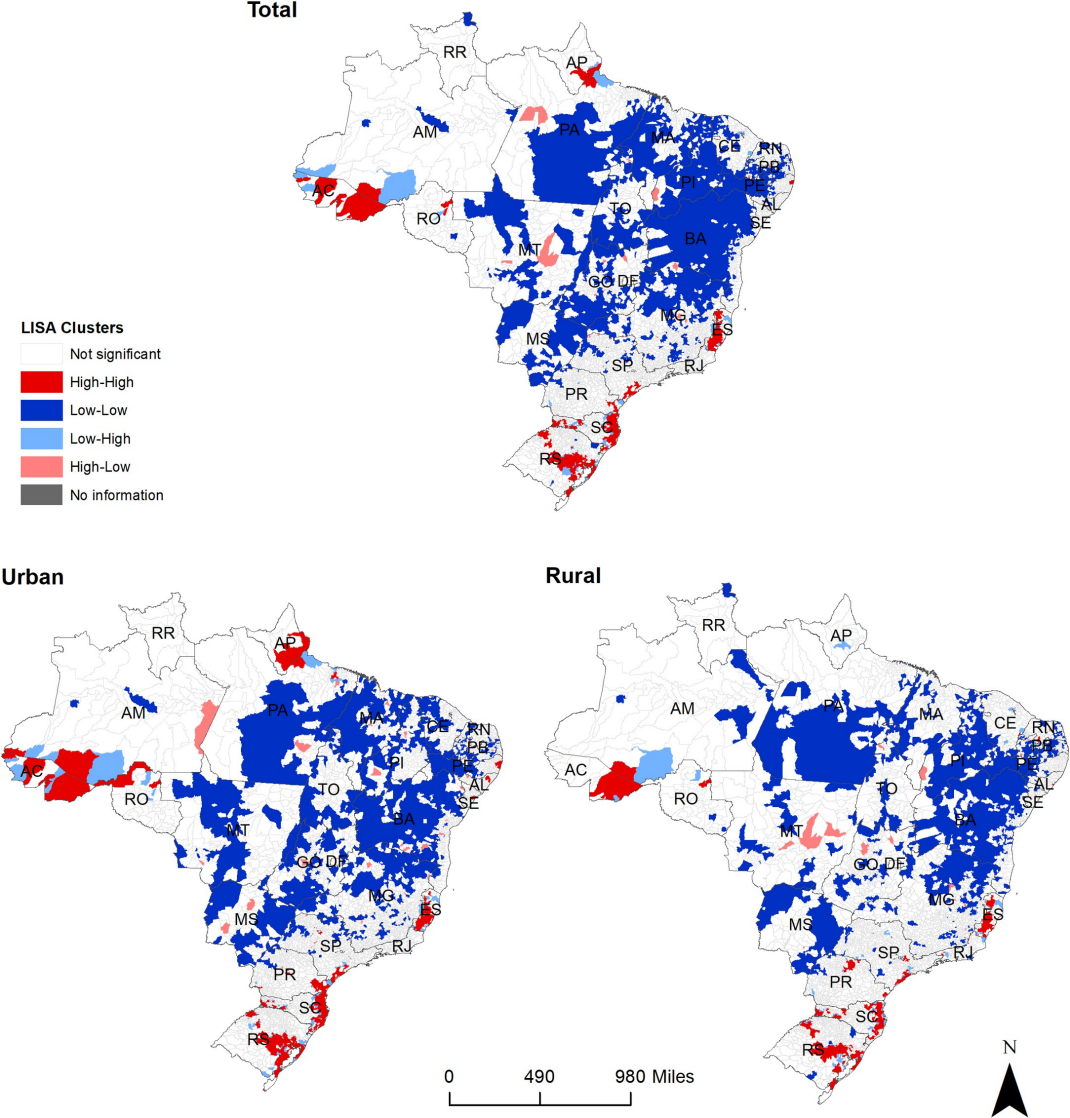
Total, urban and rural spatial clusters of leptospirosis cases in Brazil, 2000–2015.

Based on the spatial analysis, five large high-risk clusters of leptospirosis and many smaller ones were observed across Brazil (Fig 4). Similarly, in both urban and rural areas, high-risk clusters were present in the state of Espirito Santo, along the southeast coast encompassing the states of São Paulo, Paraná and Santa Catarina, and in the state of Rio Grande do Sul, in the South region. A large high-risk cluster was located in the state of Acre (North region), and this was slightly larger in urban compared to rural areas. A distinct high-risk cluster was observed in urban areas of the North region along the state of Amapá and smaller urban clusters were observed in the states of Alagoas, Pernambuco and Sergipe in the Northeast region.

## Discussion

This study of 16 years of surveillance data demonstrates that leptospirosis is an important public health problem across urban and rural areas of Brazil, with approximately 4,000 reported cases per year. Urban and rural clusters of disease were identified throughout the country. Urban clusters were located in densely populated coastal areas and in states where floods constantly occur, while rural clusters of leptospirosis were identified in regions with large agriculture activity. Outbreaks in urban centers usually occur after heavy rains, floods and other natural disasters resulting in extensive coverage by the media and direct attention of health authorities, while endemic rural areas usually have less visibility, affecting mostly poor and vulnerable populations [22, 53, 54].

Urban settings provide an ideal scenario for leptospirosis transmission, affecting particularly low-income populations living at the edge of streams and in places with poor health infrastructure, inadequate sanitation, and rodent infestations [33, 34, 37, 55, 56]. Leptospirosis epidemics related to floods, wastewater and garbage exposure have been studied in urban slums populations of São Paulo, Rio de Janeiro, Salvador and Recife [36–40]. In other parts of the world, similar reports were found in Colombia, India, Malaysia, Philippines and Sri Lanka [17, 27, 57–59]. Our study identified large clusters of disease in major urban centers where outbreaks have been reported in recent years: Santa Catarina in 2008 (997 cases) and 2011 (700 cases), São Paulo in 2011 (980 cases), Rondônia in 2014 (190 cases) and Acre in 2014 (1,196 cases) [7].

In rural areas, endemic transmission of leptospirosis has been reported in low and middle-income countries with increased risk during the warm months and rainy season [9, 19]. In China, Ecuador, India, Mexico and Sri Lanka, rural leptospirosis cases have been associated with farmers, people who have close contact with cattle and other animals around the home, and regions with greater agricultural practice [9, 21, 23, 26, 60–63]. In rural areas of Brazil, leptospirosis is not as extensively studied and information about exposure risk factors is limited. A recent study conducted in the state of Rio Grande do Sul identified possible drivers of leptospirosis related to specific ecoregions, soil and production of tobacco [20] In another study conducted in the same state by Barcellos et al., the highest incidence rates were found in the coastal sedimentary areas with low altitude and predominantly in areas with agricultural land use [41].

Urban clusters, usually associated with flood events, should be considered in national and local preparedness plans, focusing on reducing the number of severe cases and saving lives during outbreaks. Preventive strategies in rural areas require, on the other hand, strong collaboration between the public health and agriculture sectors, focusing on occupational risk factors. Due to the limited number of leptospirosis studies in rural areas of Brazil, future research should focus in understanding the disease epidemiology and risk factors in endemic rural populations and characterizing the occupation risk factors for the disease to inform decision-makers and public health professionals about the critical areas and groups for priority and targeted interventions.

Although nationally urban and rural leptospirosis incidence rates were the same (1.9 per 100,000 population), about half of the country presented higher state-specific incidence in rural areas. Regionally, higher incidence rates of the disease were observed in urban areas of the North and rural areas of the South of Brazil. The North region encompass approximately 80% of the Amazon Rainforest and includes the state of Acre, which presented the country’s highest incidence in both urban and rural areas, up to 10 times greater than the national incidence. In previous analysis from the Brazilian Ministry of Health, the government recognized the unusual high incidence of leptospirosis in the state given the increasing number of floods in Acre, specifically in 2012, 2014 and 2015, combined with inadequate sanitation and low income in the region [64–67].

Other states in the North of Brazil also had high disease incidence, portraying the unique scenario of leptospirosis in the region. Poor housing conditions along river banks with lack of access to adequate sanitation and waste collection present the perfect environment for rodent infestation and increased risk of exposure to contaminated water [65]. Previous studies recognize the importance of community education and training of healthcare professionals about the risk of leptospirosis in the Northern region throughout the year, not only during the rainy season, as well as improvements to sanitation and waste collection systems [31, 67, 68].

In the South region, the state of Rio Grande do Sul presented the highest rural incidence rate of leptospirosis in the country. Previous studies conducted in that state showed the highest incidence rates were predominantly in areas with agricultural land use, including rice and tobacco plantation [20, 41]. In the South region, floods events, combined with environmental drivers and agriculture land use provide an environment suitable for leptospirosis transmission [20, 41, 69] and this is supported by our results.

Regarding the demographic characteristics of leptospirosis cases, the results of this study are in line with previous research that shows nationally higher incidence among adult males in economically productive age groups, with notable regional differences by residence area [1]. In our study the South region had the highest incidence among males in both urban and rural areas, approximately five times higher than females in respective areas, with slightly older cases in rural settings. This demographic pattern aligns with the region’s strong economy based on subsistence agriculture and animal farming, led mostly by men between 45 and 65 years old [70]. Additional studies are needed to further understand the occupational risk factors of leptospirosis in Rio Grande do Sul to inform the development of targeted intervention strategies.

Among females, the highest incidence was detected in the urban areas of the North region suggesting possible exposure to *Leptospira* during and after flood events. In contrast, in rural areas the Northeast and Central-West regions, incidence in females was close to zero. Future studies are needed to understand the burden of leptospirosis in women, especially in regions with high rates of poverty where access to the health care system is difficult. Regional differences by race were expected due to the racial distribution across the country with higher percentage of mixed-race in the North, Northeast and Central-West regions, and white in the South and Southeast [44].

Across urban and rural areas, the most predominant exposure factor reported in all regions of the country was exposure to places with signs of rodents. Rodents play a major role in the transmission cycle and are the universal reservoirs for *Leptospira*, transferring the infection to farm animals, dogs and humans [5, 53, 71]. Rodent control activities is one of the preventive measures against leptospirosis, especially in urban settings, and can include changing the environment to reduce rodent populations (i.e., through improved sanitation and waste collection) [53, 72]. Studies are needed to measure the effectivity of rodent control activities as a preventive measure for leptospirosis in both urban and rural area.

Exposure to water/mud from floods was also identified as an important exposure factor for leptospirosis in urban and rural areas. Brazil is the most flood-prone country in the Latin America and Caribbean region and is one of the top 15 countries worldwide with the greatest population at risk for river floods [73]. Several studies conducted in Brazil, particularly in urban settings, have identified rain and floods as one of the main risk factors for leptospirosis because flooding brings the bacteria and their animal hosts into closer contact with humans [33, 35, 36, 74, 75]. In 2011, the year with the highest number of leptospirosis cases, Brazil experienced two large flood events, one in the state of Santa Catarina and one in the mountain region of the state of Rio de Janeiro [76, 77]. Temporal analysis of the relationship between rainfalls levels and leptospirosis in the state of Santa Catarina for a period of 11 years showed a positive association between the amount of rainfall and cases of the disease [69]. In the upcoming years, heavy rainfall and floods are projected to increase as a result of climate change, especially in the tropics, and this can further exacerbate the burden of leptospirosis in the future [35, 56, 78, 79].

Leptospirosis is a multifactorial disease that requires a combination of prevention and control strategies at the national, community and individual level, coupled with actions to improve the living and working conditions of the most affected populations. Prevention and control actions in both urban and rural areas should focus on reducing the risk of flooding, especially among those living at the edge of streams and in places with poor infrastructure and prone to rodent infestations. Improvements to sanitation and waste collection systems can reduce the risk of exposure to leptospirosis, particularly among those living in urban slums [34, 39, 55, 80].

Agriculture and animal farming are well-documented risk factors for leptospirosis and were identified as the main reported exposure in rural areas of Brazil. The country’s large agriculture and animal farming sectors, combined with its tropical and sub-tropical climates provide an ideal setting for transmission of leptospirosis. Studies in the Northern and Southern regions of the country have reported greater risk in subsistence farm workers, cattle farmers and areas with irrigated farming [21, 41, 42, 81].

This study has some limitations. As with many passive surveillance systems, leptospirosis in Brazil is likely underreported and misdiagnosed. Disease reporting varies across states and relies on cases reaching the healthcare system, which can cause the detection and notification of cases to be underestimated, particularly in resource poor areas of the country, the majority of which are in rural areas. Individuals with mild symptoms may not seek health care and due to the disease non-specific symptoms, leptospirosis is often misdiagnosed as other febrile illnesses like dengue and malaria, further contributing to its underreporting [53]. For these reasons, the results of our spatial cluster analysis in Central-West and Northeast states, identified as low-risk areas, should be interpreted with caution. Due to the very low number of cases reported in these regions and taking into consideration the limitations cited above, more studies are recommended to confirm the low incidence rates of leptospirosis in this part of country, especially in rural and low-income areas.

Leptospirosis’ dynamic transmission highlights the need for a One Health approach to its study and control. That is, an integrated approach that takes into account humans, animals and the environment must be used to better understand the disease and develop enhanced preventive and control strategies [2, 20, 82]. This study provides valuable information about the unique epidemiological patterns of leptospirosis in urban and rural areas of Brazil and the importance of characterizing the area-specific human-animal-environment interface. Future transdisciplinary collaborations are needed to improve our understanding of disease transmission to develop targeted strategies among high-risk groups in the One Health interface.

Although leptospirosis remains a neglected disease in many parts of the world [2, 83], Brazil is making significant efforts to prevent, detect and control the disease. In addition to sustained surveillance activities and case management, prevention and control measures should be strengthened. Activities should include increasing community awareness about the risk of exposure in urban and rural settings, training healthcare professionals to identify, treat and report the disease, as well as improving waste disposal and sanitation, coupled integrated rodent control activities. In addition, due to leptospirosis non-specific symptoms and potential misdiagnosis, the Brazilian surveillance team should strive to implement nationwide laboratory confirmation of all cases of leptospirosis. Even though leptospirosis is an epidemic-prone disease with a wide geographic distribution and among the leading zoonotic causes of morbidity and mortality worldwide [1], it is not yet considered a “tool-ready” disease for global initiatives [84]. Brazil, like many other countries where leptospirosis remains an important public health problem, needs new and improved tools for early detection and large-scale effective prevention strategies, including vaccines, to ensure the safety and health of its citizens.

## Supporting information

S1 Fig Cumulative number of cases of leptospirosis and average rainfall by month, Brazil, 2000–2015. (TIF)

S2 Fig Urban, rural and total incidence of leptospirosis per 100,000 inhabitants by state, Brazil, 2000–2015. (TIF)

S3 Fig Total, urban and rural incidence of leptospirosis per 100,000 inhabitants by county, Brazil, 2000–2015. (TIF)

S4 Fig Cases of leptospirosis by age and region in urban and rural areas, Brazil 2000–2015. (TIF)

S5 Fig Percentage of leptospirosis cases by race and region in urban and rural areas, Brazil, 2000–2015. (TIF)

S1 File Percentage of cases of leptospirosis by exposure factors and region in urban and rural areas, Brazil, 2000–2015. *Data available after 2007.
(PDF)

S1 Dataset Cases of leptospirosis analyzed, Brazil, 2000–2015. (XLS)
