# Supplementary figures and images for: Epidemiology of human leptospirosis in urban and rural areas of Brazil, 2000–2015
Source: PLoS One. 2021 Mar 4;16(3):e0247763. doi: 10.1371/journal.pone.0247763 (PMC7932126; doi:10.1371/journal.pone.0247763)

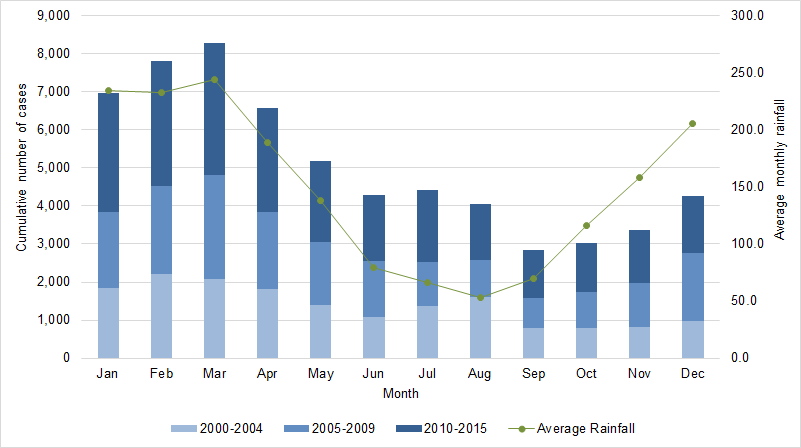

Supplement: S1 Fig — (TIF) [file pone.0247763.s001.tif]

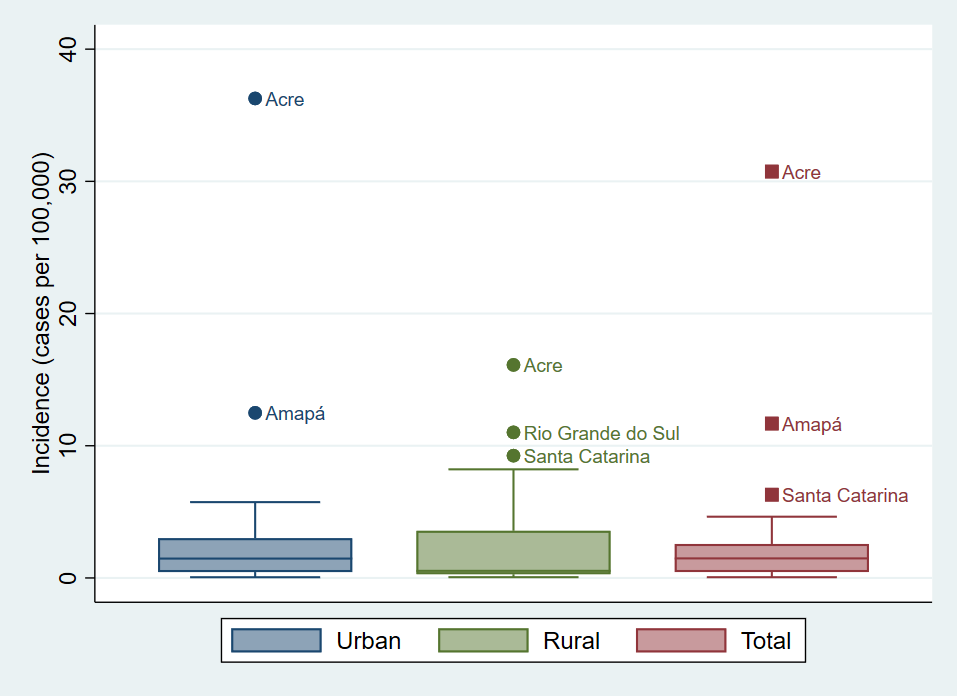

Supplement: S2 Fig — (TIF) [file pone.0247763.s002.tif]

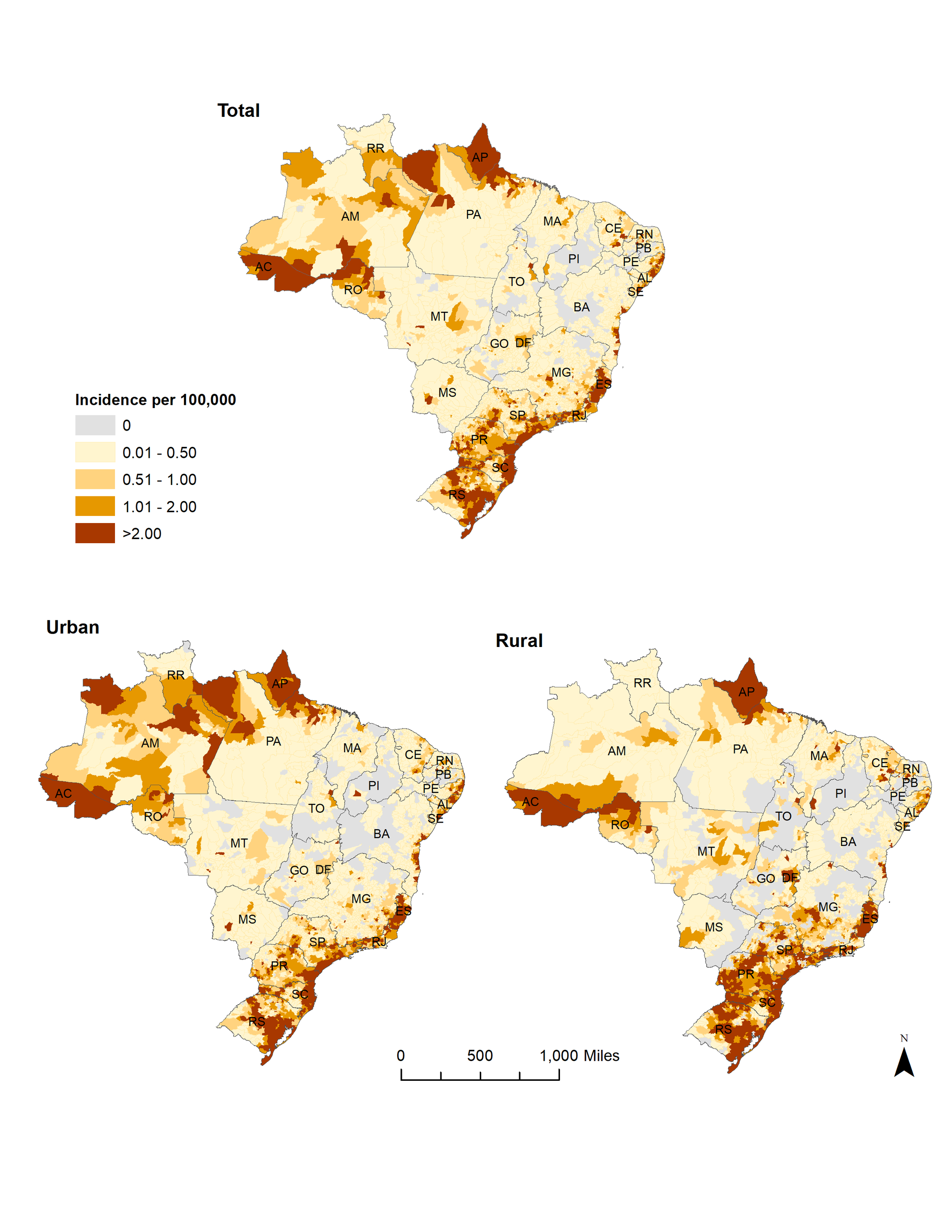

Supplement: S3 Fig — (TIF) [file pone.0247763.s003.tif]

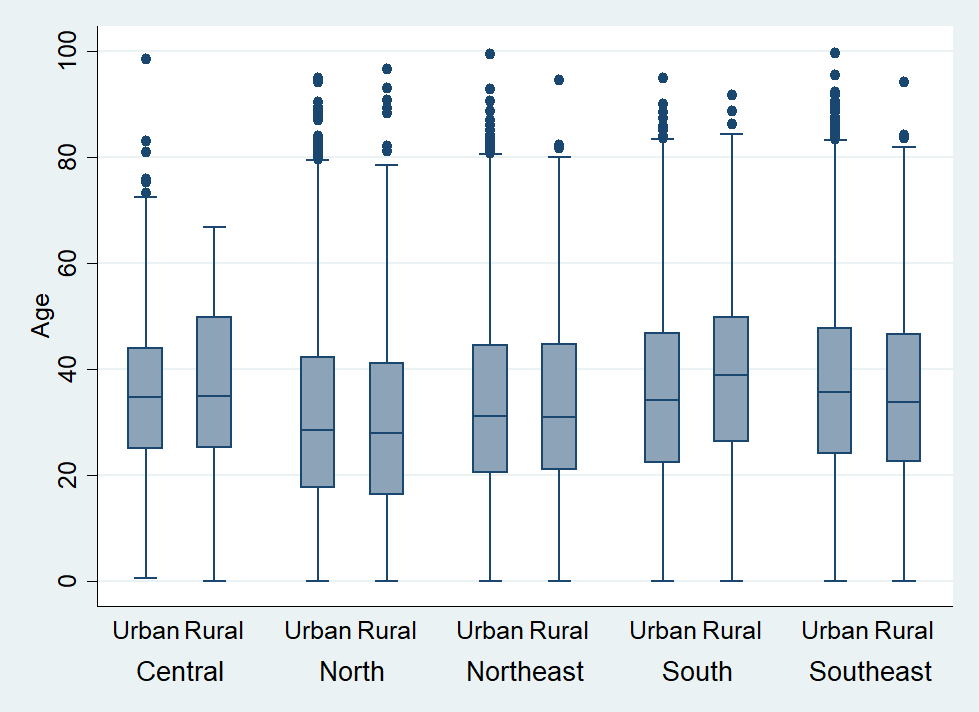

Supplement: S4 Fig — (TIF) [file pone.0247763.s004.tif]

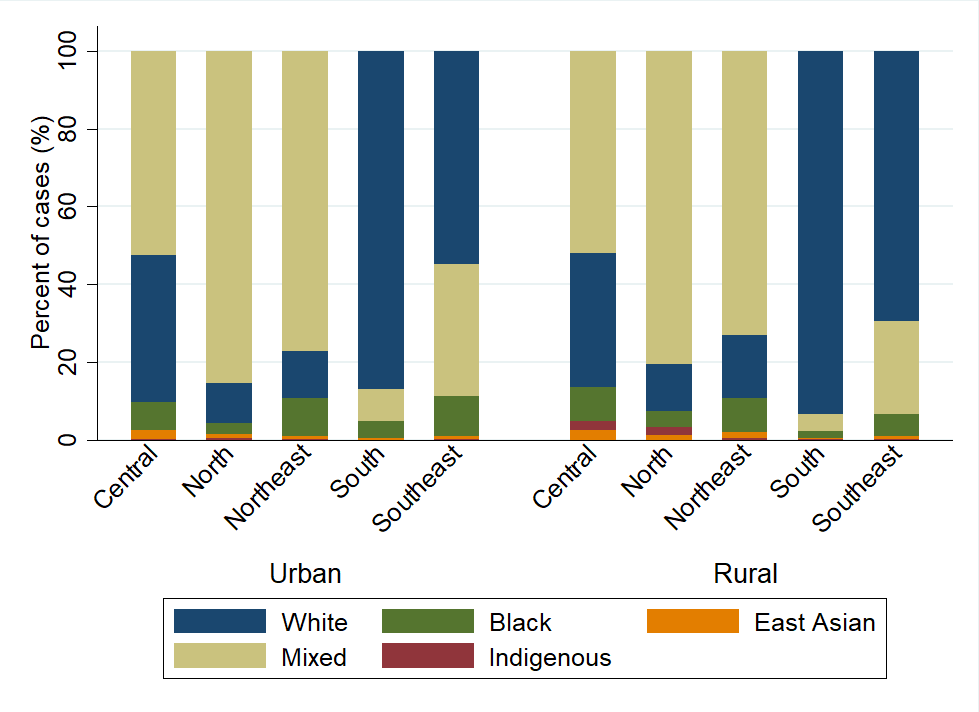

Supplement: S5 Fig — (TIF) [file pone.0247763.s005.tif]
