# Supplementary material for: Epidemiology of human leptospirosis in urban and rural areas of Brazil, 2000–2015
Source: PLoS One. 2021 Mar 4;16(3):e0247763. doi: 10.1371/journal.pone.0247763 (PMC7932126; doi:10.1371/journal.pone.0247763)
Supplement: S1 File — *Data available after 2007. (PDF) [file pone.0247763.s006.pdf]

S6 File. Percentage of cases of leptospirosis by exposure factors and region in urban and rural areas, Brazil, 2000-2015.

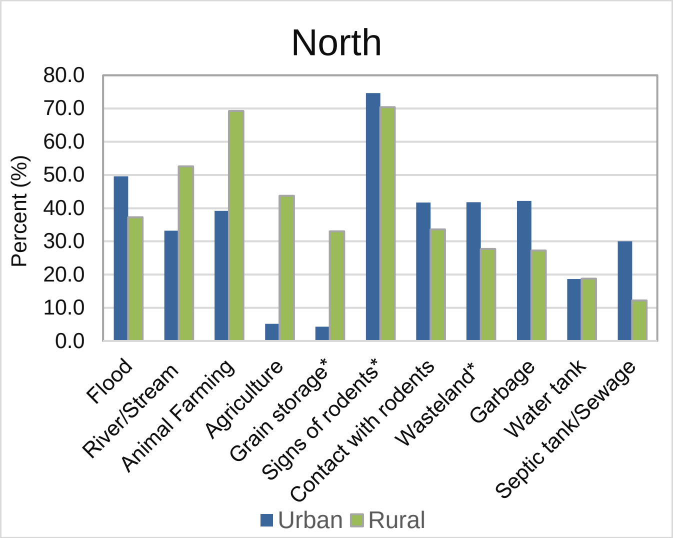

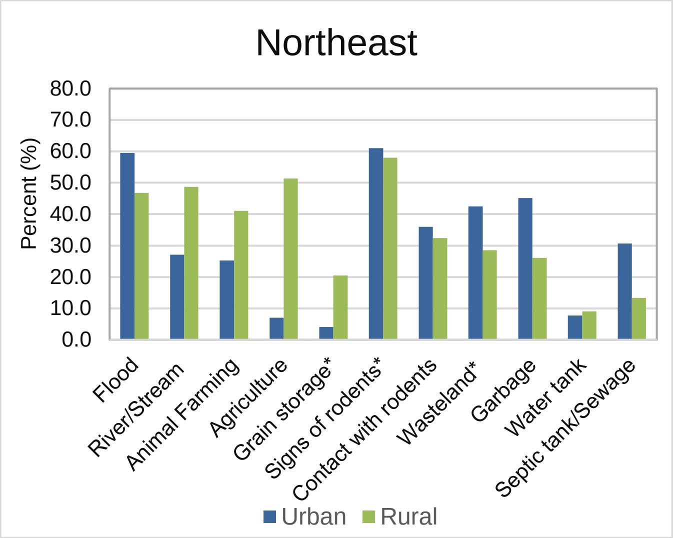

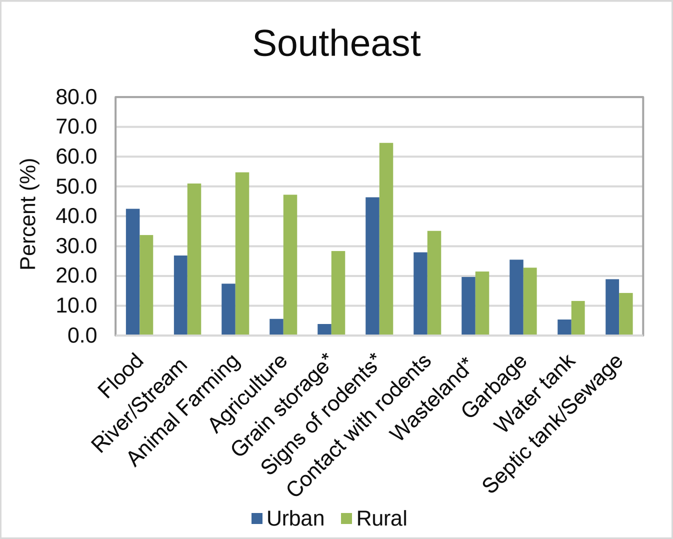

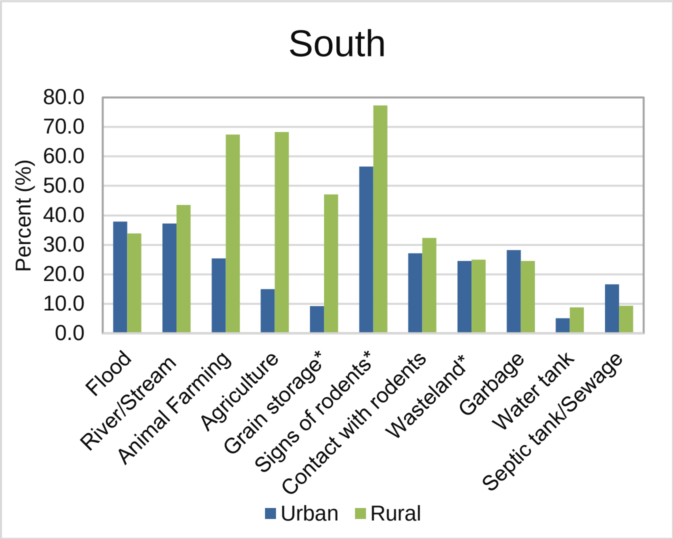

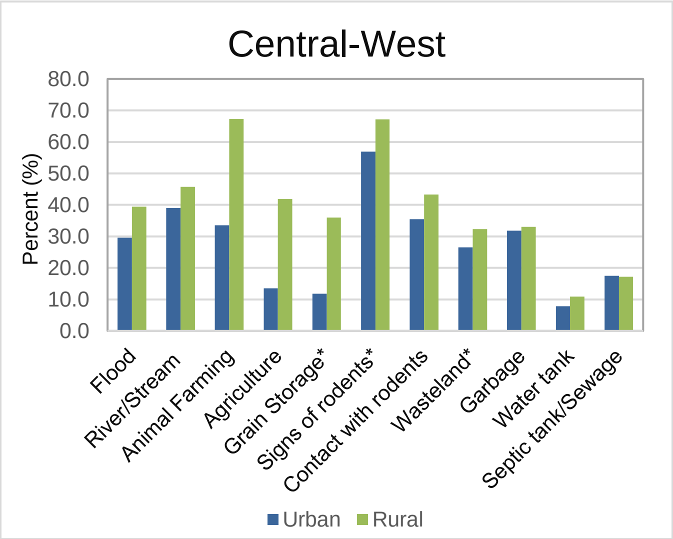

*Data available after 2007
